# The Role of PPAR****γ**** in * Helicobacter pylori* Infection and Gastric Carcinogenesis

**DOI:** 10.1155/2012/687570

**Published:** 2012-08-09

**Authors:** Jong-Min Lee, Sung Soo Kim, Young-Seok Cho

**Affiliations:** Department of Internal Medicine, Uijeongbu St. Mary's Hospital, College of Medicine, The Catholic University of Korea, Uijeongbu 480717, Republic of Korea

## Abstract

Peroxisome proliferator-activated receptor *γ* (PPAR*γ*) is a nuclear receptor that is important in many physiological and pathological processes, such as lipid metabolism, insulin sensitivity, inflammation, cell proliferation, and carcinogenesis. Several studies have shown that PPAR*γ* plays an important role in gastric mucosal injury due to *Helicobacter pylori* (*H. pylori*). As *H. pylori* infection is the main etiologic factor in chronic gastritis and gastric cancer, understanding of the potential roles of PPAR*γ* in *H. pylori* infection may lead to the development of a therapeutic target. In this paper, the authors discuss the current knowledge on the role of PPAR*γ* in *H. pylori* infection and its related gastric carcinogenesis.

## 1. Introduction

**Figure 1:**
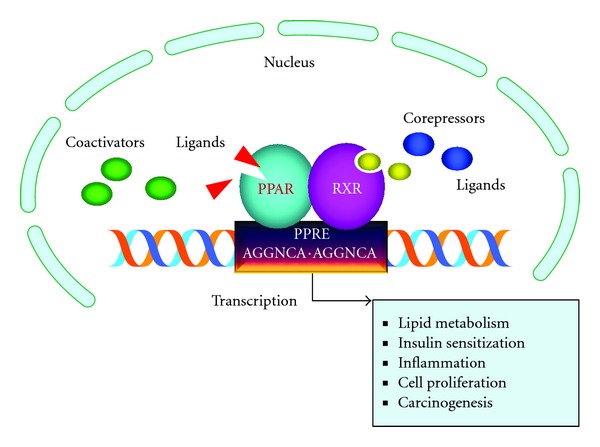
A basic mechanism of PPAR signaling. Following ligand binding, PPAR forms a heterodimer with RXR, which binds to the PPRE of target genes and regulates the transcription of these genes.

Peroxisome proliferator-activated receptors (PPARs) are ligand-activated transcription factors and members of the nuclear hormone receptor superfamily. To date, three isoforms of PPARs (PPAR*α*, PPAR*δ*/*β*, and PPAR*γ*) have been identified in mammals. PPAR forms a heterodimer with its preferential binding partner—retinoid X receptor (RXR). The function of PPAR/RXR heterodimer depends on its interactions with cofactor complexes (coactivators or corepressors). After activation by ligand, the PPAR/RXR heterodimer binds to specific DNA response elements called peroxisome proliferator response elements (PPREs) of the target genes. This results in transcription regulation of these genes (Figure 1) [1]. PPARs play a significant role in regulation of fatty acid oxidation and glucose utilization [2]. PPAR*γ* was originally identified as a differentiation transcription factor for adipose tissue [3]. In addition, PPAR*γ* is involved in the control of inflammation and glucose metabolism and participates in the processes of cellular proliferation, differentiation, and apoptosis [4]. Natural ligands for PPAR*γ* are 15-deoxy-Δ^12,14^-prostaglandin J_2_ (15d-PGJ_2_) and various polyunsaturated fatty acids [5, 6]. The insulin sensitizing thiazolinediones, which are selective ligands of the nuclear transcription factor PPAR*γ*, were the first drugs used to treat insulin resistance in patients with type II diabetes [7].

*Helicobacter pylori *(*H. pylori*) infection is the main etiologic agent in gastric inflammation, and longstanding infection with this organism is linked to gastric cancer [8]. Based on epidemiological studies, the risk of gastric cancer conferred by *H. pylori* has been estimated to be 75% [9]. Although the mechanism of *H. pylori*-induced carcinogenesis is still being investigated, inflammation is the strongest risk factor in the carcinogenic process [10], because it affects host responses such as epithelial cell proliferation and apoptosis [9]. PPAR*γ* may be involved in the regulation of gene expression associated with inflammation and cancer. This paper reviews current knowledge of the role of PPAR*γ* in *H. pylori* infection and its related gastric carcinogenesis.

## 2. PPAR**γ** Expression in *H. pylori* Infection

**Figure 2:**
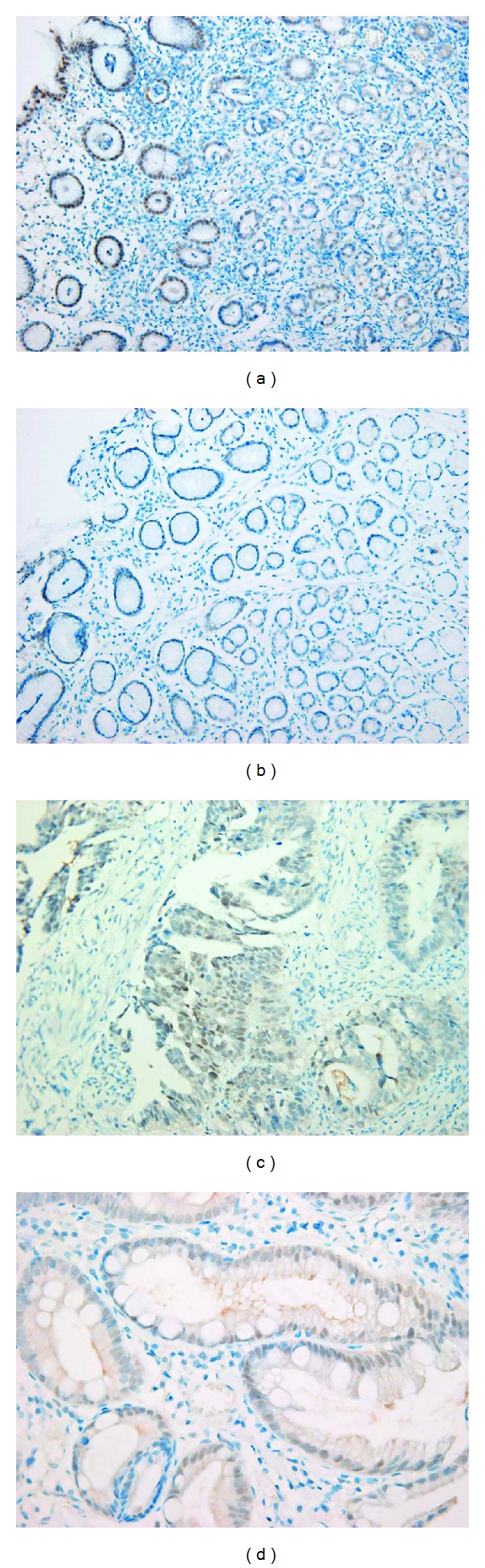
PPAR*γ* protein expression in various tissues. Strong nuclear staining in gastric epithelial cells of *H. pylori*-infected patients (a) and weak nuclear staining in *H. pylori*-negative subjects (b). PPAR*γ* protein is also expressed in the nucleus of tumor cells in gastric adenocarcinoma (c) and in noncancerous tissue with intestinal metaplasia adjacent to cancer tissue (d).

PPAR*γ* is predominantly expressed in adipose tissue, intestinal epithelium, monocytes and macrophages, the retina, skeletal muscle, and lymphoid organs [1]. Braissant et al. demonstrated PPAR*γ* expression in the adult rat gastric mucosa by *in situ* hybridization and immunohistochemistry [11]. Several studies have found that PPAR*γ* expression increases during *H. pylori* infection [12–14]. First, Konturek et al. demonstrated that PPAR*γ* gene and protein expression were significantly higher in the gastric mucosa of *H. pylori*-positive gastric cancer patients than in *H. pylori*-negative healthy controls [12]. In addition, *H. pylori* eradication significantly reduced PPAR*γ* expression. We demonstrated previously that PPAR*γ* expression, identified by immunohistochemistry, was mostly detected in the nucleus of the foveolar epithelial cells in gastric mucosa and the intensity of PPAR*γ* expression was significantly higher in the 18 patients with *H. pylori*-associated chronic gastritis than in the 21 *H. pylori*-negative patients (Figure 2) [13]. However, there was no correlation between the numbers of neutrophils and PPAR*γ* expression in the two groups. Haruna et al. reported results similar to ours [14]. In this study, cyclooxygenase-2 (COX-2) and PPAR*γ* mRNA expression in the gastric mucosa of children were found to be increased with *H. pylori* infection. The expression of COX-2, which plays an important role in inflammation, carcinogenesis, and development, is regulated by a negative feedback loop mediated through PPAR*γ* [15]. Overexpression of both PPAR*γ* and COX-2 was detected in the gastric mucosa of Mongolian gerbils infected with *H. pylori* [16]. Taking these findings together, enhanced PPAR*γ* expression in gastric mucosa infected with *H. pylori* may have anti-inflammatory and cytoprotective effects.

## 3. The Role of PPAR**γ** Activation ****in *H. pylori* Infection

Several studies have demonstrated that PPAR*γ* has an overall anti-inflammatory effect [2, 17]. Molecular mechanisms include inhibition of signaling pathways regulating expression of proinflammatory genes (e.g., nuclear factor (NF)-*κ*B) and stress-kinase pathways [17]. Gupta et al. demonstrated that PPAR*γ* activation suppresses *H. pylori*-induced apoptosis in gastric epithelial cells and that this effect is mediated by direct inhibition of *H. pylori*-induced NF-*κ*B activation [18]. *H. pylori* lipopolysaccharide (LPS), a component of the outer membrane, is a potent virulence factor for mucosal inflammatory changes, and its mechanism is mediated by increased proinflammatory cytokine production, excessive nitric oxide (NO) and PG generation, and epithelial cell apoptosis [19, 20]. B. L. Slomiany and A. Slomiany reported that PPAR**γ**activation by ciglitazone inhibits gastric mucosal inflammation and that this effect is mediated by reduced apoptosis, mucosal PGE2 generation, expression of COX-2, and inducible nitric oxide synthase (NOS-2) [20]. In addition, ciglitazone impedes the inhibition of *H. pylori* LPS on gastric mucin synthesis, an effect likely dependent on the activation of the extracellular signal-related kinase (ERK) pathway by phosphatidylinositol 3-kinase (PI3K) [21]. These findings suggest that PPAR**γ**activation may provide therapeutic benefits in *H. pylori*-associated gastric inflammation.

 The transactivation of epidermal growth factor receptor (EGFR) is strongly linked to *H. pylori* infection, gastric epithelial hyperplasia, and gastric atrophy [10]. It depends on genes in the *cag* pathogenicity island, secreted proteins, and host factors such as TLR4 and NOD1 [22]. The biological responses to EGFR transactivation include increased proliferation, reduced apoptosis, altered cell polarity, and enhanced migration [10]. Although the underlying mechanism involved in the differential activation by ciglitazone of the EGFR/Erk mitogen activated protein kinases (MAPK) pathway is not well understood, this effect can be mediated by activation of Erk, an event requiring Src-kinase-dependent EGFR transactivation [23]. Ciglitazone has been shown to suppress *H. pylori* LPS inhibition of gastric mucin synthesis mediated by Src-kinase-dependent EGFR transactivation [24].

## 4. The Role of PPAR**γ** in *H. pylori*-Related ****Gastric Carcinogenesis

Although the incidence of gastric adenocarcinoma is decreasing, it remains the second most common cause of cancer-related mortality worldwide [25]. There are large variations in the incidence and death rates among racial and ethnic groups, and the gastric cancer incidence and death rate are twice as high as in Asian American/Pacific islanders compared with Caucasians, reflecting an increased prevalence of chronic *H. pylori* infection [26]. Although gastric cancer treatments are continuously improving, the prognosis for this disease is poor and the survival rate is less than 40% even after curative resection and adjuvant chemotherapy [27].

Although the involvement of PPAR*γ* in the development of cancer in various tissues remains controversial, PPAR*γ* activation has antitumorigenic effects due to its antiproliferative and prodifferentiation activities [1]. Several *in vitro* studies have found that PPAR*γ* activation results in cell cycle arrest and/or apoptosis of gastric cancer cells [28–32]. First, Takahashi et al. demonstrated that a human gastric cancer cell line, MKN45, expressed PPAR*γ* mRNA and protein, and that PPAR*γ* activation inhibited cell growth and induced apoptosis in gastric cancer cells [28]. Sato et al. used immunohistochemistry to show that the PPAR*γ* protein is expressed in surgically resected specimens from well-, moderately, and poorly differentiated gastric adenocarcinomas as well as in noncancerous gastric mucosa with intestinal metaplasia [29]. The results of our study of PPAR*γ* protein expression in gastric adenocarcinoma and normal mucosa with intestinal metaplasia adjacent to cancer were consistent with Sato's results (Figure 2) [13]. However, recent studies have reported that redistribution of PPAR*γ* expression occurs in human gastric adenocarcinoma [33–35]. The immunohistochemical staining pattern of PPAR*γ* is nuclear in the normal gastric mucosa but primarily cytoplasmic in intestinal metaplasia (IM) [33]. The high cytoplasmic-to-nuclear expression ratio of PPAR*γ* decreases as the differentiation stage changes from IM to adenoma, and to well-, moderately-, and poorly-differentiated cancers. PPAR*γ* is expressed primarily in the nucleus in metastatic gastric cancer [34]. Burgermeister et al. demonstrated that the molecular mechanisms of PPAR*γ* redistribution include interaction with Ras/mitogen activated protein kinases (MAPKs) such as caveolin-1 (Cav1) and docking protein 1 (Dok1) [35].

The inhibitory effect of PPAR*γ* on gastric cancer may be attributed to various mechanisms. Ligand-induced activation of PPAR*γ* was found to inhibit c-MET (an important protooncogene-encoding receptor for hepatocyte growth factor) [36] and expression of cyclin D1 [37] and COX-2 [31], induce expression of p27 [38], p21 [32], and p53 [39], and suppress gastrin (a potent cancer cell growth promoting factor) [40]. An *in vivo* animal study determined that heterozygous (PPAR*γ* ^+/−^) knockout mice were more susceptible to N-methyl-N-nitrosourea-induced gastric carcinoma than homozygotes (PPAR*γ* ^+/+^), but troglitazone only reduced the incidence of gastric cancer in homozygotes [41].

Konturek et al. reported that expression of tissue PPAR*γ*, tissue levels of proinflammatory cytokines (IL-1*β* and IL-8), and plasma gastrin concentrations were significantly higher in *H. pylori*-positive gastric cancer compared to *H. pylori*-negative controls, but *H. pylori* eradication reduced these parameters [12]. These findings suggest that these parameters could be implicated in *H. pylori*-related gastric carcinogenesis. An *in vitro* study found that *H. pylori* infection downregulates the expression of p27 in gastric epithelial cells even in the absence of inflammation [42]. Reduced expression of p27 is found in *H. pylori*-associated intestinal metaplasia and *H. pylori* eradication reverses the aberrant expression of p27 [43]. Low p27 protein expression has been reported to be associated with increased expression of p27-specific F-box protein Skp2 and *H. pylori* eradication reverses the aberrant expression of p27 and Skp2 protein [44]. However, p27 and Skp2 mRNA levels were unaffected by *H. pylori* eradication, suggesting that *H. pylori* may influence cell cycle progression and carcinogenesis post-translational effects on specific gene expression. We have shown that rosiglitazone inhibited the growth of *H. pylori*-infected gastric epithelial cells [45]. These effects of rosiglitazone were associated with decreased Skp2 expression, thereby promoting p27 accumulation in *H. pylori*-infected gastric epithelial cells.

## 5. PPAR**γ** Polymorphism in *H. pylori*-Related ****Gastric Carcinogenesis

A common PPAR*γ* polymorphism, a C to G substitution in exon B, results in a proline to alanine exchange at codon 12 (Pro12Ala) [46]. Functionally, this Ala variant has been reported to show decreased binding to the response element and a lower capacity for activating target genes [47]. PPAR*γ* polymorphism (Pro12Ala) has been found to be associated with various diseases including type II diabetes, cardiovascular disease, and several types of cancer [48]. Pro12Ala polymorphism lowers the risk of diseases in colorectal cancer and type II diabetes [49]. These results could be partly explained by the etiological link between type II diabetes and colorectal cancer. On the contrary, several studies have demonstrated that the Pro12Ala polymorphism is associated with the high risk of gastric adenocarcinoma [48, 50–53]. Liao et al. reported that the carriage of G phenotype or Ala allele in codon 12 of PPAR*γ* was associated with a 2.5-fold increase in the risk of noncardia gastric cancer in Chinese, and that the risk was higher when this polymorphism was combined with *H. pylori* infection [50]. A recent meta-analysis suggested that carriers of Pro12Ala have a 2.31-fold (95% CI = 1.59–3.36, *P*_heterogeneity_ = 0.941) increased gastric cancer risk [48]. Considering that PPAR*γ* activation inhibits the growth of gastric cancer cells, these results suggest that gastric carcinogenesis may have a different genetic background than colorectal carcinogenesis.

The role of Pro12Ala polymorphism in peptic ulcer disease remains controversial. Prasad et al. showed that patients with Pro12Ala polymorphism were susceptible to peptic ulcer disease in the presence of *H. pylori* infection [52]. Meanwhile, Bazargani et al. found no significant increase in the risk of peptic ulcer formation among patients with Pro12Ala polymorphism [53]. This discrepancy may be due to the role of other bacterial virulence factors and/or host factors.

## 6. Conclusions

In this paper, we focused on the role of PPAR*γ* in *H. pylori* infection and its related gastric carcinogenesis. PPAR*γ* suppresses inflammation in *H. pylori* infection and tumor growth in gastric cancer. Emerging evidence indicates that mutations in PPAR*γ* may play a crucial role in the development of noncardia gastric cancer in *H. pylori*-infected patients. Therefore, further studies are needed to investigate modulation of PPAR*γ* as an effective therapy for chemoprevention and treatment of inflammation in *H. pylori* infection and gastric cancer.
